# Computed tomography and magnetic resonance imaging in Brazil: an epidemiological study on the distribution of equipment and frequency of examinations, with comparisons between the public and private sectors

**DOI:** 10.1590/0100-3984.2023.0094-en

**Published:** 2024-04-14

**Authors:** Camila de Almeida Costa Alencar, Dante Claudino de Oliveira, Aylla Batista Moreira Teixeira, Lívia Maria Goes Lemos, Raquel Cristina Saldanha Quesado, Ionara Maria de Almeida Santos, Carolina Freitas Lins

**Affiliations:** 1 Escola Bahiana de Medicina e Saúde Pública (EBMSP), Salvador, BA, Brazil; 2 Clínica Delfin Medicina Diagnóstica, Salvador, BA, Brazil

**Keywords:** Tomography, X-ray computed, Magnetic resonance imaging, Health information systems, Public sector, Private sector, Tomografia computadorizada, Ressonância magnética, Sistemas de informação em saúde, Setor público, Setor privado

## Abstract

**Objective:**

To compare information on highly complex radiological procedures—computed tomography (CT) and magnetic resonance imaging (MRI)—between the public and private health care systems, across the five regions of Brazil, in terms of the numbers of radiological devices and examinations performed, between 2015 and 2021.

**Materials and Methods:**

This was a descriptive time series analysis of secondary data in the public domain, available from the Information Technology Department of the Brazilian Unified Health Care System, an entity of the Brazilian National Ministry of Health (NMH) that is responsible for collecting and storing health-related information in Brazil. The analysis included the numbers of CT and MRI scanners; the volumes and types of examinations; the type of institution (public or private); the regions of the country; and the years (2015 to 2021).

**Results:**

Progressive increases in the numbers of CT and MRI devices, as well as in the volumes of examinations, were observed over the years in all regions of the country. The private sector showed higher rates of equipment acquisition and of growth in the number of examinations. However, the public health care system did not reach the equipment targets set by the NMH, whereas the private health care system surpassed those targets. A greater number of examinations were performed in the private sector than in the public sector.

**Conclusion:**

During the period evaluated, the public health care system did not meet the equipment or examination targets recommended by the NMH, in any of the regions of the country, unlike the private health care system, which exceeded both in all of the regions.

## Abstract (pt)

**Objetivo:**

Comparar informações sobre procedimentos radiológicos de alta complexidade – tomografia computadorizada (TC) e ressonância magnética (RM) –, considerando o número de aparelhos e o quantitativo de exames nas esferas pública e privada nas cinco regiões brasileiras entre 2015 e 2021.

**Materiais e Métodos:**

Trata-se de um estudo descritivo de série temporal que utilizou dados secundários do Departamento de Informática do Sistema Único de Saúde, órgão do Ministério da Saúde (MS) responsável pela coleta e armazenamento das informações relacionadas à saúde no Brasil. Analisamos os números de aparelhos e de exames de TC e RM, considerando os tipos de aparelhos e exames, instituição (pública ou privada), região brasileira e ano (2015 a 2021).

**Resultados:**

Houve aumento de aparelhos e exames de TC e RM em todas as regiões ao longo dos anos. A esfera privada apresentou maior aquisição desses aparelhos e crescimento no número de exames. O sistema público não atingiu o número de aparelhos preconizado pelo MS, enquanto o sistema privado superou a recomendação. Observou-se maior número de exames na esfera privada quando comparada à pública.

**Conclusão:**

O sistema público não atendeu aos números de aparelhos e exames realizados preconizados pelo MS, diferentemente da esfera privada, em todas as regiões no período estudado.

## INTRODUCTION

Diagnostic medicine is crucial in health care, encompassing a variety of examinations, including radiological imaging studies^(1)^. Although the foundations of this area date back to the beginnings of medical science, it was thanks to technological advances and the deepening of disciplines such as physics, chemistry, and pathophysiology that it reached its current level of precision and applicability. Thus, diagnostic medicine has come to account for a significant portion of the health professional labor market and is expanding rapidly^(2)^.

The Brazilian *Sistema Único de Saúde* (SUS, Unified Health Care System) aims to guarantee full, universal, free access to health care, including diagnostic medicine services, to all citizens^(3)^. Therefore, radiological examinations are included among the procedures offered by the SUS, which categorizes them by the degree of complexity^(4)^: medium complexity—radiography and ultrasound; and high complexity—computed tomography (CT) and magnetic resonance imaging (MRI). Despite being charged with providing these procedures by the federal government, the SUS is not always able to meet the high demand for them^(3)^. Consequently, the concepts of complementary health care, which combines efforts from the private sector with those from the public sector, and supplementary health care, which refers to services provided via private health care plans, regulated and supervised by the Brazilian *Agência Nacional de Saúde Suplementar* (ANS, National Health Insurance Agency), have been incorporated^(5)^.

The Information Technology Department of the SUS (DATASUS) is an organ of the Secretariat for Strategic and Participatory Management of the Brazilian National Ministry of Health (NMH), responsible for collecting, processing, storing, and disseminating data on health care services in Brazil, including radiological procedures of high complexity. The DATASUS obtains information from health care systems and institutions affiliated with the SUS, making it available to the general public and serving not only as a repository of information but also as a valuable tool for analyzing and understanding public health in the country^(3,6,7)^. Those data influence decisions made by health care managers and professionals, allowing the identification of specific needs, the detection of trends, strategic planning, the appropriate allocation of resources, and evaluation of the results of implemented policies. In addition, making access to information open and free allows researchers and society in general to conduct studies, contributing to the body of scientific knowledge and monitoring of the health care system in Brazil^(8)^.

Although the SUS provides most radiological procedures free of charge, it is estimated that there is inequality among the regions of Brazil, as well as between the public and private sectors, in terms of the distribution of equipment and the availability of examinations^(9)^. Given this context, the objective of the present study was to compare the public and private sectors in all five regions of Brazil, on the basis of the information available in the DATASUS regarding highly complex radiological procedures (CT and MRI), considering the distribution and quantity of radiological equipment in use, as well as the volume of examinations carried out, between 2015 and 2021.

## MATERIALS AND METHODS

This was a descriptive study employing secondary data from the DATASUS. The requirement for research ethics committee analysis was waived because the study used public-domain data, without any personal or identifiable information about the individuals involved. The study covered the entire territory of Brazil, including the five regions of the country (north, northeast, central-west, south, and southeast), for the period from 2015 to 2021.

### Information systems

Highly complex equipment was defined as CT and MRI devices in operation, data on which are available from the National Registry of Health Care Facilities for each region of Brazil. Data regarding the number of examinations were collected from the *Sistema de Informação Ambulatorial* (SIA, Outpatient Information System) and *Sistema de Informação Hospitalar* (SIH, Hospital Information System) of the SUS. Data relating to supplementary health care were also analyzed.

Information from the National Registry of Health Care Facilities, the SIA, and the SIH are available from the DATASUS (https://datasus.saude.gov.br/), whereas data on supplementary health care are available on the ANS website (https://www.gov.br/ans/pt-br). To calculate proportions and coefficients, we used the population estimates available from the *Instituto Brasileiro de Geografia e Estatística* (IBGE, Brazilian Institute of Geography and Statistics) for the 2015–2021 period^(10)^.

### Data analysis

The processing, analysis, and organization of data, as well as the creation of graphs and tables, were carried out by two researchers (a fifth-year medical student and a radiologist with 17 years of experience), using the Microsoft Office Excel program, version 10.

To analyze the geographic distribution of CT and MRI devices and the annual numbers of examinations performed via the SUS, we followed the recommendations outlined in NMH Ordinance no. 1,631/2015^(11)^ and the NMH booklet “Parameters for Scheduling Health Care Activities and Services of Medium to High Complexity Via the SUS”^(12)^.

The calculations used in order to determine the quantity of CT and MRI equipment, as well as the number of MRI examinations, needed to serve the population of each region are shown in equations 1, 4, and 5. In NMH Ordinance no. 1,631/2015^(11)^, there is no defined parameter for the number of CT examinations required, nor was that information found in any other NMH document.


Eq. 1 $$T=\left(10\times estimatednumberofinhabitantsinaregioninagivenyear\right)/1\times 10^{6}$$

Eq. 2 SR=(totalpopulation×30)/1,000

Eq. 3 N=SR/5,000

Eq. 4 $$NR=\left(numberofinhabitantsofaregion\times 6\right)/1\times 10^{6}$$

Eq. 5 $$SR=\left(estimatednumberofinhabitantsinaregioninagivenyear\times 30,000\right)/1\times 10^{6}$$

### Annual number of CT scanners required per region

On the basis of NMH Ordinance no. 1,631/2015^(11)^, which calls for 10 CT scanners per million inhabitants, the following equation was employed:

### Annual number of MRI scanners required per region

According to NMH Ordinance no. 1,631/2015^(11)^, the required number of MRI scanners is six per million inhabitants. The productivity of an MRI scanner is 5,000 examinations per year, and the estimated number of scans needed is 30 per 1,000 inhabitants:

where *SR* is the (number of) scans required.

To calculate the number of MRI scanners needed, the following equation was employed:

where *N* is the number of scanners needed and *SR* is the (number of) scans required.

That culminated in an equation for calculating the quantity of MRI scanners needed per region:

where *NR* is the number of scanners needed for the region.

### Number of CT examinations required per year and region

Because NMH Ordinance no. 1,631/2015^(11)^ does not provide information on the number of CT examinations required to serve the population per year and region, it was not possible to carry out a comparative analysis of those parameters.

### Number of MRI examinations required per year and region

According to NMH Ordinance no. 1,631/2015^(11)^, an estimated 30,000 MRI examinations are needed per million inhabitants in Brazil. To calculate the annual number of MRI scans required per region, we used the following equation:

where *SR* is the (number of) scans required.

### Year-to-year percentage change

The annual percentage change was calculated for the population and for the number of highly complex examinations, as well as for the numbers CT and MRI scanners. That change was calculated with the following equation:


Eq. 6 APC=[(secondyearvalue−firstyearvalue)/(firstyearvalue)]×100

where *APC* is the annual percentage change.

### Average percentage growth

The average percentage growth for the 2015–2021 period was calculated for the population and for the number of highly complex examinations, as well as for the numbers of CT and MRI scanners. That growth was calculated with the following equation:


Eq. 7 APG=(∑annualgrowthpercentages/numberofpercentages)×100

## RESULTS

### Estimated population by region

According to IBGE estimates^(10)^, there were population increases in all regions of Brazil over the years evaluated, with an average growth rate of 0.72% during the 2015–2021 period (Table 1).

**Table 1:** Estimated resident population of Brazil, by region and year.

Year	Region					Total	∆A%	AG%
	Southeast	Northeast	South	North	Central-west			
2015	85,745,520	56,560,081	29,230,180	17,472,636	15,442,232	204,450,649	—	0.72
2016	86,356,952	56,915,936	29,439,773	17,707,783	15,660,988	206,081,432	0.80	
2017	86,949,714	57,254,159	29,644,948	17,936,201	15,875,907	207,660,929	0.77	
2018	87,521,700	57,576,309	29,843,748	18,158,149	16,086,896	209.186.802	0.73	
2019	88,072,407	57,883,049	30,036,030	18,373,753	16,293,774	210.659.013	0.70	
2020	88,601,482	58,174,912	30,221,606	18,583,035	16,496,340	212,077,375	0.67	
2021	89,107,377	58,453,160	30,398,904	18,786,300	16,694,717	213,440,458	0.64	

Source: IBGE^(10)^. ∆A%, annual percentage change; AG%, average percentage growth during the 2015–2021 period.

### Number of CT and MRI scanners needed in each region

The numbers of CT and MRI devices required in order to serve the population of each region of Brazil satisfactorily are shown in Table 2. An increase in the quantity of CT and MRI scanners needed was observed over the years in all regions (Table 2), following the trend of population growth.

**Table 2:** Total numbers of CT and MRI scanners needed in Brazil, by region and year.

Year	CT					MRI				
	Southeast	Northeast	South	North	Central-west	Southeast	Northeast	South	North	Central-west
2015	857	566	292	175	154	514	339	175	105	93
2016	864	596	294	177	157	516	341	177	106	94
2017	869	573	296	179	159	522	344	178	108	95
2018	875	576	298	182	161	525	345	179	109	97
2019	881	579	300	184	163	528	347	180	110	98
2020	886	582	302	186	165	532	349	181	111	99
2021	891	585	304	188	167	535	351	182	113	100

Source: NMH Ordinance no. 1,631/2015.

### Number of CT and MRI scanners in use in the public and private sectors in each region

During the 2015–2021 period, there were increases in the numbers of CT and MRI devices in use in all regions (Table 3), and those increases were more pronounced in the private sector. In all regions and years, the proportion of devices in use was highest in the private sector, which accounted for more than 60% of the equipment required for highly complex radiological examinations.

**Table 3:** Spatial distribution of CT and MRI equipment in use in the public and private sectors in Brazil, by region, from 2015 to 2021.

CT	Public sector								Private sector							
	Southeast n (%)	Northeast n (%)	South n (%)	North n (%)	Central-west n (%)	Total	∆%	AG%	Southeast n (%)	Northeast n (%)	South n (%)	North n (%)	Central-west n (%)	Total	∆%	AG%
2015	246 (12.8)	155 (22.5)	46 (6.9)	39 (17.6)	43 (11.7)	529	—	7.6	1319 (68.6)	483 (70.2)	425 (63.4)	171 (77)	289 (78.7)	2687	—	7.0
2016	263 (13.4)	119 (16.5)	49 (6.8)	42 (18.2)	41 (10.3)	514	-2.8		1353 (68.9)	514 (71.1)	455 (63.4)	175 (75.8)	319 (79.8)	2816	4.8	
2017	270 (12.8)	137 (17.2)	54 (7.1)	45 (18)	44 (9.6)	550	7.0		1477 (69.9)	565 (71.1)	485 (64.2)	190 (76)	369 (80.6)	3086	9.6	
2018	271 (12)	151 (18)	57 (7)	49 (17.6)	46 (9.5)	574	4.4		1584 (70.4)	595 (70.4)	527 (64.8)	209 (72.2)	392 (81)	3307	7.2	
2019	285 (12)	160 (17.9)	56 (6.3)	50 (17.8)	47 (8.6)	598	4.2		1641 (66.8)	635 (70.9)	589 (66.6)	208 (74)	459 (83.6)	3532	6.8	
2020	351 (14.1)	196 (20.4)	64 (6.9)	60 (19)	61 (9.9)	732	22.4		1665 (66.8)	659 (68.5)	619 (66.5)	229 (72.7)	507 (81.9)	3679	4.2	
2021	370 (13.8)	224 (20.8)	62 (6.3)	73 (19.4)	78 (11.3)	807	10.2		1800 (67.4)	733 (68.1)	662 (66.8)	274 (72.9)	556 (80.3)	4025	9.4	
MRI																
2015	58 (5.6)	16 (5.5)	10 (2,7)	11 (10.9)	4 (2.4)	99	—	10.8	803 (77.4)	247 (84.6)	267 (2.7)	84 (83.2)	148 (89.7)	1549	—	7.8
2016	69 (6.4)	21 (6.4)	10 (2,6)	11 (10.0)	3 (1.8)	114	15.2		832 (77.6)	280 (84.8)	288 (73.5)	93 (84.5)	151 (88.8)	1644	6.1	
2017	70 (6.0)	33 (8.6)	12 (2,9)	14 (10.8)	5 (2.3)	134	17.5		928 (78.9)	319 (83.1)	311 (74.2)	109 (82.3)	193 (89.4)	1860	13.1	
2018	67 (5.3)	43 (10.3)	15 (3,3)	18 (12.2)	6 (2.5)	149	11.2		996 (79.4)	346 (82.6)	336 (74.0)	121 (82.3)	211 (89.0)	2010	8.1	
2019	70 (5.2)	50 (10.6)	14 (2,9)	19 (12.6)	6 (2.3)	159	6.7		1061 (79.1)	382 (81.3)	363 (75.5)	122 (80.8)	233 (90.3)	2161	7.5	
2020	76 (5.5)	52 (10.8)	15 (2,9)	18 (11.4)	8 (2.6)	169	6.3		1083 (78.5)	393 (81.7)	388 (74.9)	130 (82.3)	274 (89.9)	2268	5.0	
2021	77 (5.3)	60 (11.4)	16 (2,9)	20 (11.1)	9 (2.8)	182	7.7		1146 (79.1)	426 (81.0)	415 (75.6)	150 (83.3)	292 (89.3)	2429	7.1	

Source: NMH – DATASUS (https://datasus.saude.gov.br/). ∆A%, annual percentage change; AG%, average percentage growth during the 2015–2021 period.

The proportion of CT scanners in use was higher in the private sector, ranging from 63.4% (in the southern region in December 2015) to 83.6% (in the central-west region in December 2019). In the public sector, the proportion of CT scanners in use ranged from 6.8% (in the southern region in December 2016) to 20.8% (in the northeastern region in December 2021).

**Figure 1:**
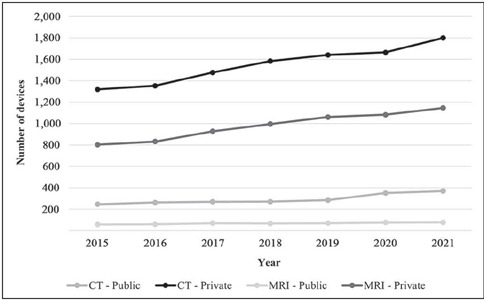
Quantity of CT and MRI scanners in the public and private system in the southeastern region.

The numbers of MRI scanners in use, in the public and private sectors, were highest in the southeastern region. Overall, the proportion of MRI devices in use was higher in the private sector, ranging from 73.0% (in the southern region in December 2015) to 90.3% (in the central-west region in December 2019). The proportion of MRI devices in use in the public sector ranged from 1.8% (in the central-west region in December 2016) to 12.6% (in the northern region in December 2019). Figure 1 compares the numbers of CT and MRI devices in use in the public and private sectors in the southeastern region. In the public sector, the average percentage growth was 7.6% for CT scanners and 10.8% for MRI scanners, compared with 7.0% and 7.8%, respectively, in the private sector.

Although the annual percentage growth was higher in the public sector, the target numbers of scanners, as defined in NMH Ordinance no. 1,631/2015^(11)^, were not met in the public sector in any region of the country during the period under study (Table 4). In the private sector, the NMH target for CT scanners was met in all regions. For CT scanners in the public sector, the average percentages of the NMH target were quite similar among regions, being lowest in the southern region, whereas in the private sector, those percentages were lowest in the northern and northeastern regions. For MRI scanners, the private sector met the NMH target in all regions except the northeastern region, whereas the target was not met in the public sector in any of the regions, the percentages being lowest in the southern and central-west regions (Table 4).

**Table 4:** Percentages met of the targets defined for CT and MRI equipment in the public and private sectors, according to the parameters outlined in NMH Ordinance No. 1,631/2015, and the averages of those percentages for the 2015–2021 period.

CT	Public sector					Private sector				
	Southeast (%)	Northeast (%)	South (%)	North (%)	Central-west (%)	Southeast (%)	Northeast (%)	South (%)	North (%)	Central-west (%)
2015	28.70	27.39	15.75	22.29	27.92	153.91	97. 71	145.55	85.34	187.66
2016	30.44	19.97	16.67	23.73	26.11	156.60	98.87	154.76	86.24	203.18
2017	31.07	23.91	18.24	25.14	27.67	169.97	106.15	163.85	98.60	232.08
2018	30.97	26.22	19.13	26.92	28.57	181.03	114.84	176.85	103.30	243.48
2019	32.35	27.63	18.67	27.17	28.83	186.27	113.04	196.33	109.67	281.60
2020	39.62	33.68	21.19	32.26	36.97	187.92	123.12	204.97	113.23	307.27
2021	41.53	38.29	20.39	38.83	46.71	202.02	145.74	217. 76	125.30	332.93
M	33.53	28.15	18.58	28.05	31.83	176.82	103.10	180.01	114.21	255.46
MRI										
2015	11.28	4.72	5.71	10.48	4.30	156.23	72.86	152.57	80.00	159.14
2016	13.37	6.16	5.65	10.38	3.19	161.24	82.11	162.71	87.74	160.64
2017	13.41	9.59	6.74	12.96	5.26	177.78	92.73	174.72	100.93	203.16
2018	12.76	12.46	8.38	16.51	6.19	189.71	100.29	187. 71	111.01	217.53
2019	13.26	14.41	7.78	17. 27	6.12	200.95	110.09	201.67	110.91	237.76
2020	14.29	14.90	8.29	16.22	8.08	203.57	112.61	214.36	117.12	276.77
2021	14.39	17.09	8.79	17.70	9.00	214.21	121.37	228.02	132.74	292.00
M	13.25	11.33	7.33	14.50	6.02	186.24	98.87	188.82	105.78	221.00

Source: NMH – DATASUS (https://datasus.saude.gov.br/). M, mean of the annual percentages of the targets set.

### Number of CT and MRI examinations required

Recommended numbers of CT examinations needed in order to serve the population were not addressed in NMH Ordinance no. 1,631/2015^(11)^. The recommended numbers of MRI examinations for each region are shown, by year, in Table 5.

**Table 5:** Number of MRI examinations needed in order to serve the population of each region of Brazil.

Year	Southeast	Northeast	South	North	Central-west	Total
2015	2,572,366	1,696,802	876,905	524,179	463,267	6,133,519
2016	2,590,709	1,707,478	883,193	531,233	469,830	6,182,443
2017	2,608,491	1,717,625	889,348	538,086	476,277	6,229,828
2018	2,625,651	1,727,289	895,312	544,744	482,607	6,275,604
2019	2,642,172	1,736,491	901,081	551,213	488,813	6,319,770
2020	2,658,044	1,745,247	906,648	557,491	494,890	6,362,321
2021	2,637,221	1,753,595	911,967	563,589	500,842	6,403,214

Source: NMH Ordinance no. 1,631/2015.

### Number of CT and MRI examinations performed in the public and private sectors

Tables 6 and 7 show that there were increases in the annual numbers of CT and MRI examinations performed via the public and private health care systems, respectively. However, during the period analyzed, the private health care system produced 20–80% more CT scans (depending on the year of analysis) than did the SUS.

**Table 6:** CT and MRI examinations in the public sector in Brazil from 2015 to 2021, by setting, region, and year.

Examination	Setting	Southeast	Northeast	South	North	Central-west	Total	∆A%	AG%
CT									
2015	Outpatient	1,271,139	373,769	149,872	135,510	147,298	3,624,501	—	13.3
	Inpatient	787,068	219,917	375,892	51,930	112,106			
2016	Outpatient	1,346,117	438,659	169,194	134,471	146,574	3,898,861	7.6	
	Inpatient	821,078	243,003	418 ,164	57,420	124,181			
2017	Outpatient	1,448,813	487,599	199,823	150,368	151,057	4,225,912	8.4	
	Inpatient	864,860	267,985	457,570	63,025	134,812			
2018	Outpatient	1,617,974	603,349	221,136	190,254	177,841	4,746,185	12.3	
	Inpatient	946,572	297,228	473,819	66,709	151,303			
2019	Outpatient	1,723,110	682,354	271,965	200,839	223,339	5,264,167	10.9	
	Inpatient	1,051,033	344,917	526,483	73,256	166,871			
2020	Outpatient	1,939,155	668,974	320,038	259,434	265,735	6,097,281	15.8	
	Inpatient	1,310,520	411,511	605,065	101,003	215,846			
2021	Outpatient	2,334,702	902,732	413,649	310,769	387,491	7,618,721	25.0	
	Inpatient	1,592,693	534,718	755,500	128,102	258,365			
MRI									
2015	Outpatient	215,556	29,444	22,150	21,095	5,968	414,239	—	13.3
	Inpatient	55,410	21,939	35,627	2,354	4,696			
2016	Outpatient	219,945	39,995	27,557	26,804	3,379	444,062	7.2	
	Inpatient	57,167	21,020	40,160	3,123	4,912			
2017	Outpatient	240,999	49,864	33,655	32,654	6,541	503,295	13.4	
	Inpatient	60,427	23,714	47,232	3 ,174	5,035			
2018	Outpatient	268,850	77,769	49,186	33,763	6,315	585,325	16.3	
	Inpatient	64,233	25,792	51 , 617	2,426	5,374			
2019	Outpatient	288,305	105,315	72,405	36,463	7,830	674,608	15.3	
	Inpatient	71,660	27,372	55,069	4,036	6,153			
2020	Outpatient	251,130	88,761	62,390	19,065	8,576	595,603	-11.7	
	Inpatient	74,832	26,296	52,609	4,236	7,708			
2021	Outpatient	297,174	128,317	82,051	29,522	15,178	731,627	25.0	
	Inpatient	82,325	28,813	53,766	5,574	8,907			

Source: NMH – SIA/SUS, SIH/SUS, and ANS. ∆A%, annual percentage change; AG%, average percentage growth during the 2015–2021 period.

**Table 7:** CT and MRI examinations in the private sector, from 2015 to 2021.

Examination	Sector					Total	∆A%	AG%
	Self-management	Physician cooperative	Philanthropic organization	Group medicine	Health insurance company			
TC								
2015	737,649	2,131,723	103,323	1,727,442	1,934,674	6,634,811	6.6	6,4
2016	786,991	2,280,114	120,090	1,883,028	2,000,731	7,070,954	1.7	
2017	786,286	2,412,265	114,633	2,088,633	1,788,413	7,190,230	2.7	
2018	927.101	2,655,113	121,396	2,215,510	1,467,756	7,386,876	3.7	
2019	944,313	2,803,590	149,713	2,463,543	1,298,379	7,659,538	-3.8	
2020	877,447	3,111,304	138,508	2,251,446	987,547	7,366,252	27.4	
2021	1,113,543	4,235,570	187,013	2,787,194	1,059,909	9,383,229	6.6	
RM								
2015	806,473	2,246,656	80,505	1,585,843	1,791,700	6,511,177	8.8	4,2
2016	840,782	2,443,979	109,448	1,846,670	1,846,107	7,086,986	4.5	
2017	841,904	2,539,008	149,716	2,069,345	1,806,840	7,406,813	6.7	
2018	977,725	2,733,888	159,371	2,094,971	1,878,512	7,904,467	5.5	
2019	1,034,872	2,844,075	244,500	2,354,308	1,860,654	8,338,409	-23.7	
2020	768,572	2,440,436	113,322	1,867,064	1,174,451	6,363,845	23.1	
2021	917,694	3,064,225	145,758	2,465,236	1,241,372	7,834,285	8.8	

Source: NMH – SIA/SUS, SIH/SUS, and ANS. ΔA%, annual percentage change; AG%, average percentage growth during the 2015–2021 period.

**Figure 2:**
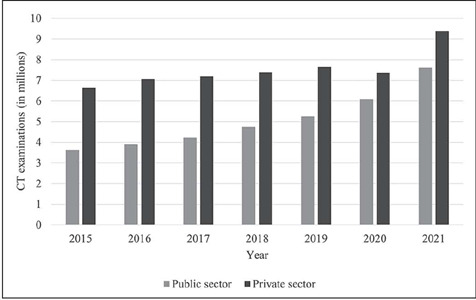
CT examinations in the public and private sectors, from 2015 to 2021.

**Figure 3:**
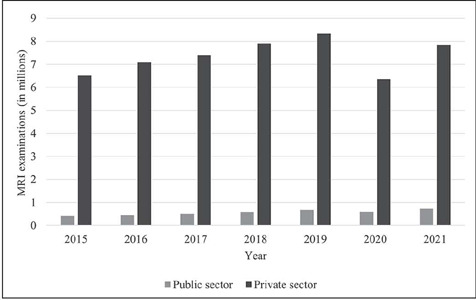
MRI examinations in the public and private sectors, from 2015 to 2021.

The number of MRI examinations performed annually was also greater in the private sector than in the public sector. Notably, the total numbers of MRI examinations performed via the SUS did not reach the recommended numbers stipulated in NMH Ordinance no. 1,631/2015^(11)^. Although the NMH has not stipulated recommended numbers of CT examinations, the data show that many more CT scans were performed in the private sector than in the public sector in all of the years studied, as illustrated in Figure 2. The recommended number of MRI examinations was not reached by the public health care system in any region of the country. However, the private system exceeded that number in all the years analyzed, as indicated in Figure 3.

## DISCUSSION

In this study, we have compared the availability of CT and MRI equipment and examinations between the public and private sectors, in all regions of Brazil, between 2015 and 2021. The most striking finding was the lack of compliance of the public health care system in relation to government guidelines, with the numbers of devices available and numbers of examinations performed being greater in the private health care system, in all regions of the country.

Similar, previous studies have addressed only one imaging method^(13)^, have covered CT and MRI but only in one Brazilian state^(14)^, have compared only the distribution of equipment^(15)^, have evaluated only the public health care system^(13,15)^, or have been restricted to only one imaging method in only one specific geographic area^(16)^. However, they all produced results similar to those of the present study, either by showing unequal distribution of equipment and examinations between the public and private sectors, with a predominance in the private sector^(13,14,17)^, or by highlighting the fact that, in most regions of Brazil, the public health care system has not met the goals set by the NMH, for CT or for MRI^(14,15,16)^. In the present study, we have provided an up-to-date overview of the distribution of CT and MRI devices in Brazil, as well as the number of examinations, in the public and private sectors, comparing them with the target values established in NMH Ordinance no. 1,631/2015^(11)^, in all regions of the country.

Studies conducted prior to the advent of NMH Ordinance no. 1,631/2015^(11)^ have also shown that the availability of equipment is greater in the private sector than in the public sector^(13,15,17)^, highlighting the fact that the SUS has not obtained the number of devices recommended by the NMH in most regions of Brazil^(14)^. In this context, it is worth remembering that, within the SUS, the accessibility of a procedure decreases as the complexity of the procedure increases^(17)^. Our findings corroborate those data by demonstrating that, even after the implementation of NMH Ordinance no. 1,631/2015^(11)^, the deficits in relation to highly complex radiological examinations persist in the SUS.

The challenges in Brazil include limited geographic access to essential health services via the SUS and the concentration of wealth in certain locations^(18)^. Many SUS users live in underserved areas, which requires them to travel long distances to access health resources^(14,18)^. Our data underscore that inequality by demonstrating a greater concentration of equipment and examinations in the southeastern region of the country, where much of the wealth is concentrated. In addition, the majority of the Brazilian population does not participate in the private health care system^(19)^, in which there is a greater concentration of equipment and through which greater numbers of examinations are performed, a fact that heightens the inequality in health care access^(17,20,21)^. In the present study, it was evident that the majority of CT and MRI scanners in Brazil are in service in the private sector. Therefore, the majority of the population faces difficulties in accessing highly complex examinations, either because of geographic barriers to undergoing those via the SUS or because of a lack of financial resources to resort to the private sector (complementary or supplementary health care).

It is of note that only 28.5% of Brazilian citizens have private health insurance^(22)^ . However, as observed in our research, the private sector owns most of the equipment and performs most of the highly complex (CT and MRI) examinations. Therefore, the majority of the available resources are accessed by less than 30% of the population and the majority of the population depends on the public health care system, which has many fewer resources. That results in a discrepancy between the demand for highly complex examinations and the real capacity to access such examinations. According to Federal Law no. 8080, the SUS is responsible for formulating the policy on medicines, equipment, biologic agents, and other material of interest to health, as well as for participating in their production^(23)^. However, government investment in public health in Brazil is likely to be insufficient^(24)^, especially with recent reductions in public investment in all social spheres, including health^(25)^. The financial management of the SUS, influenced by neoliberal economic policy, has contributed to a reduction of social capital, as have the increase in privatization and the support for large corporations^(26)^. Although there have been increases in the numbers of CT and MRI scanners acquired by the SUS, as well as in the numbers of CT and MRI examinations performed therein, the public health care system has not kept pace with the private system. During the period analyzed, the public sector, in isolation, was unable to meet the demand for highly complex procedures according to the recommendations of NMH Ordinance no. 1,631/2015^(11)^.

According to the IBGE, an annual average, in Brazilian reals (R$), of R$2,035.60 per capita is invested in family and institutional expenses related to health care in the private sector, compared with R$1,349.60 per capita invested in the public health care sector^(10)^. In 2019, the average per capita health expenditure in Organisation for Economic Co-operation and Development (OECD) countries exceeded $4,000. The United States led with $11,000. Switzerland spent two-thirds of that amount, whereas Norway and Sweden spent a bit more than half of what was spent in the United States. Brazil is among the smallest investors in the OECD, investing less than half of the average but more than nations such as Indonesia and India^(27)^. Spending on health in Brazil has reached 9.6% of the gross domestic product, with 5.8% going to the private sector, more than double the OECD average, which was 2.3%. As for spending on public health, Brazil appears near the bottom of the list of OECD countries, allocating only 3.8% of its gross domestic product to the SUS. Albeit ahead of Mexico, Brazil is behind European countries and other Latin American countries such as Colombia and Chile^(10,27)^. Although our study does not directly evaluate health spending in the public and private sectors, our analysis of the increases in the numbers of scanners and the total numbers of examinations performed over the years allows us to verify the discrepancy in spending between the two sectors.

In the United States, most people obtain health insurance through their employers or separately from private companies^(28,29)^, although some access health care services through government programs for specific populations, such as the elderly and low-income individuals^(29,30)^. In Canada, however, the government guarantees universal access to health care for all citizens^(31)^. In those two countries, imaging methods such as CT and MRI are becoming more widely used, representing some of the main services paid for by health insurance^(28,32,33)^. Although those methods facilitate the diagnosis and treatment, they can also generate costs and risks, such as overdiagnosis^(28,34)^. In this context, it is estimated that 30% of the imaging examinations performed in the United States and Canada are unnecessary^(28,35)^. In Brazil, which has a public health care system similar to that of Canada, there is also a trend toward excessive imaging examinations^(36)^. Our study demonstrated that much higher numbers of tests were performed in the private sector than in the public sector, raising the question of whether those values were related to overdiagnosis, similar to what has been reported for other countries^(28,34,35,36)^. However, the databases used in the present study do not provide information about the indications for the examinations, and further studies are needed in order to confirm our assumption.

Our study has some limitations: a) the lack of information in the DATASUS about the number of joint public-private initiatives existing in the country and the amount of equipment and examinations managed by such initiatives, although that does not invalidate the results of our analyses; b) the fact that the NMH does not establish parameters regarding the annual number of CT examinations needed to serve the population, as well as that the ANS does not provide the number of examinations performed in the private sector by region; c) the fact that our analysis of the equipment covered in our study was quantitative, rather than qualitative, making it impossible to compare factors such as time of use and the state of maintenance of the devices; d) the fact that although philanthropic organizations are technically within the private sector, they must allocate at least 60% of their services to the SUS^(37)^. However, neither the DATASUS nor the ANS delineate the proportion of examinations carried out by philanthropic organizations within the scope of the SUS. Despite these limitations, our data can guide public bodies in managing the availability of resources, as well as demonstrating existing gaps in the DATASUS where there is room for improvement. Future perspectives include the incentive to carry out other, more in-depth studies on the topic.

## CONCLUSION

In all regions of Brazil and during all of the years evaluated, the public sector had lower numbers of devices and of highly complex radiological (CT and MRI) examinations than did the private sector. In addition, in the SUS, there was a notable lack of compliance with the parameters outlined in NMH Ordinance no. 1,631/2015^(11)^.
